# Seagrass blue carbon spatial patterns at the meadow-scale

**DOI:** 10.1371/journal.pone.0176630

**Published:** 2017-04-27

**Authors:** Matthew P. J. Oreska, Karen J. McGlathery, John H. Porter

**Affiliations:** Department of Environmental Sciences, University of Virginia, Charlottesville, Virginia, United States of America; University of Alabama, UNITED STATES

## Abstract

Most information on seagrass carbon burial derives from point measurements, which are sometimes scaled by meadow area to estimate carbon stocks; however, sediment organic carbon (C_org_) concentrations may vary with distance from the meadow edge, resulting in spatial gradients that affect the accuracy of stock estimates. We mapped sediment C_org_ concentrations throughout a large (6 km^2^) restored seagrass meadow to determine whether C_org_ distribution patterns exist at different spatial scales. The meadow originated from ≤1-acre plots seeded between 2001 and 2004, so we expected C_org_ to vary spatially according to the known meadow age at sample sites and with proximity to the meadow edge. Applying spatial autoregressive models allowed us to control for spatial autocorrelation and quantify the relative effects of edge proximity and age on C_org_ concentrations. We found that edge proximity, not age, significantly predicted the meadow-scale C_org_ distribution. We also evaluated relationships between C_org_ and a variety of specific explanatory variables, including site relative exposure, shoot density, sediment grain size, and bathymetry. Factors known to affect carbon burial at the plot-scale, such as meadow age and shoot density, were not significant controls on the meadow-scale C_org_ distribution. Strong correlations between C_org_, grain size, and edge proximity suggest that current attenuation increases fine-sediment deposition and, therefore, carbon burial with distance into the meadow. By mapping the sediment C_org_ pool, we provide the first accurate quantification of an enhanced carbon stock attributable to seagrass restoration. The top 12 cm of the bed contain 3660 t C_org_, approximately 1200 t more C_org_ than an equal area of bare sediment. Most of that net increase is concentrated in a meadow area with low tidal current velocities. Managers should account for the effects of meadow configuration and current velocity when estimating seagrass blue carbon stocks. Our results suggest that a large, contiguous meadow should store more blue carbon than an equal area of small meadow patches.

## Introduction

Seagrass meadows are highly productive ecosystems [1] that bury organic carbon [2,3], making them sinks in the global carbon cycle [4]. Bed accretion from canopy particle trapping, seston burial, and sediment anoxia [5,6,7] facilitate high sediment carbon burial rates in seagrass meadows [8]. However, the global disappearance of meadows [9] causes bed erosion [10] and the loss of accumulated sediment carbon [11], a significant carbon stock in many meadows [12]. Organic carbon (C_org_) oxidation in degraded seagrass beds potentially releases 0.05 to 0.33 Pg CO_2_ back to the atmosphere each year [13]. Efforts to finance seagrass restoration through the sale of ‘blue carbon’ offset-credits aim to reverse this trend [14,15].

Despite significant work on seagrass sediment carbon dynamics [16,17,18], little is known about how sediment C_org_ accumulates spatially in seagrass beds [19]. If the resulting stock is non-uniformly distributed throughout a meadow, multiplying an average cm^3^-scale concentration obtained from individual plots by total meadow area [2, 20] would yield an inaccurate meadow-scale (ha or km^2^) stock estimate. In a restored meadow that is expanding, C_org_ concentrations may vary spatially simply because younger meadow areas have had less time to accumulate C_org_. However, other meadow and landscape-scale factors might also cause sediment C_org_ to exhibit spatial variability, including hydrodynamics [21,22], canopy structure [22,23,24], and environmental gradients, such as bathymetry [24,25,26]. Some of these factors may give rise to C_org_ spatial gradients, especially relative to the boundary between the meadow and bare subtidal areas, hereafter referred to as the ‘edge’ [27].

**Fig 1:**
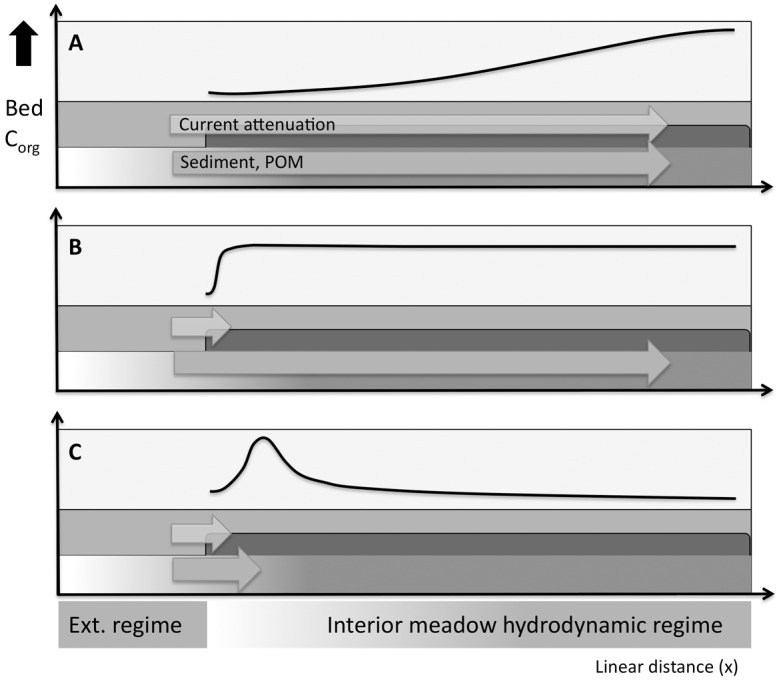
Hypothetical sediment C_org_ spatial distributions (black lines) relative to a seagrass meadow edge. These hypotheses assume (A) increasing current attenuation with distance into the meadow, (B) attenuation over a short distance and high suspended sediment availability, and (C) attenuation with low suspended sediment availability.

Several different C_org_ spatial gradients might result from current flow effects relative to the meadow edge (Fig 1). Seagrass canopies affect near-bed shear stress, which affects sediment accumulation [28,29,30]. Particle settling facilitates the burial of both seagrass detritus and external particulate organic matter [7]. At the meadow edge, higher turbulence [23] and wave energy [30] may cause erosion, potentially preventing C_org_ accumulation relative to interior sites. Progressive canopy attenuation of waves and tidal currents [30,31,32] may result in increasing C_org_ burial with distance into the meadow (Fig 1A). If, however, canopies attenuate shear stress to a constant level over short distances (perhaps <0.5 m, cf. [33,34]), the ‘edge zone’ may be narrow and have a negligible impact on the total C_org_ stock (Fig 1B). Alternatively, external sediment supply may decrease with distance as particles settle out [32], causing sediment accumulation to peak and then decrease towards the meadow center (Fig 1C). Canopy filtration of external particulate organic matter [35] and deflection of incoming wave energy [36,37] may result in more C_org_ burial near the edge.

Some empirical evidence now exists for seagrass sediment C_org_ concentration variability at patch- and regional-scales [24,27], but there is a lack of empirical support to link site and process-based studies with spatial patterns at the meadow-scale. Few seagrass studies sample over whole meadow areas and most do not account for spatial autocorrelation (for example, [24]), thereby limiting their ability to identify statistically significant controls on C_org_ accumulation at spatial scales ≥1 km.

The existence of meadow-scale C_org_ spatial gradients would have implications for blue carbon finance for seagrass restoration. A framework now exists for allocating carbon dioxide equivalent (CO_2_e) offset-credits to restoration projects. This framework, Verified Carbon Standard methodology VM0033 [38], requires that managers stratify project areas to account for spatial variability when estimating blue carbon stocks (cf. [39] for information on stratification). If zones of higher and lower C_org_ accumulation exist within meadows, managers must identify these zones (i.e. strata) and scale average C_org_ concentrations within each zone separately to generate a whole-meadow stock estimate.

The restored *Zostera marina* (eelgrass) meadow in South Bay, Virginia, provides a unique opportunity to investigate sediment C_org_ spatial patterns at the meadow-scale. This mature meadow has a well-documented restoration and expansion history [40,41,42,43], allowing us to consider C_org_ accumulation time (i.e. site meadow age) as an independent variable potentially affecting the C_org_ spatial distribution. Eelgrass seeds were broadcast in South Bay in 0.5 and 1-acre seed plots from 2001–2004 [40], which later coalesced into a single, contiguous meadow encompassing >6 km^2^ [43], making it the world’s largest restored seagrass meadow. This meadow is accumulating C_org_ from both eelgrass and allochthonous sources relative to bare sites [3,42,44]. Wind-generated shear stress is a dominant control on sediment suspension throughout this coastal bay system [45,46]. The meadow attenuates both wave and tidal currents, thereby affecting suspended sediment concentrations and sediment accretion rates relative to bare areas [3,47]. We expected to find sediment C_org_ spatial gradients related to edge proximity, in addition to a C_org_ distribution pattern related to meadow expansion. Spatial gradients related to differences in relative location and meadow age may be attributable to co-varying factors known to affect sediment accumulation at individual meadow sites, especially shoot density [26,42].

## Methods

### Study area

**Fig 2:**
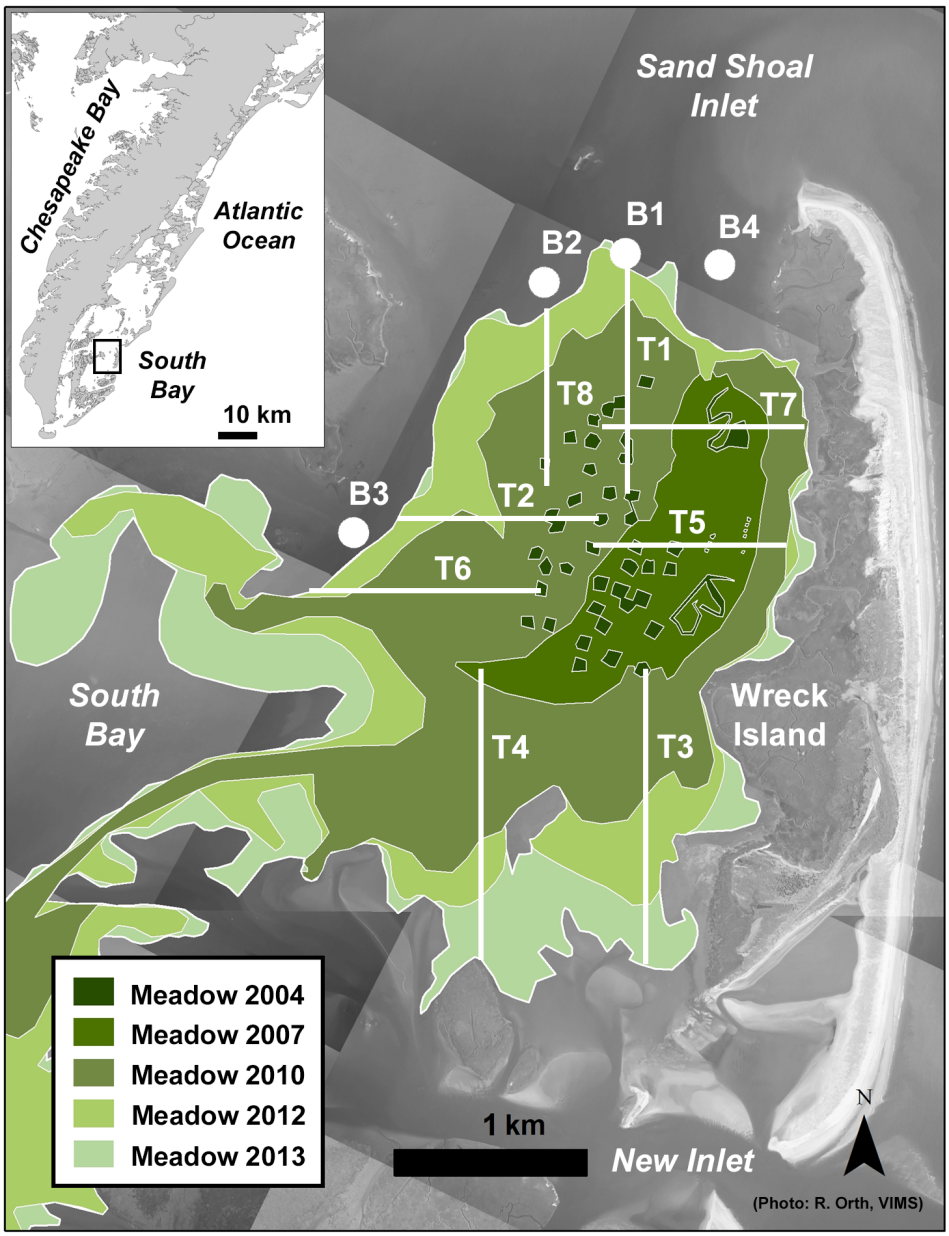
The restored South Bay eelgrass meadow showing its expansion history and sampling transects. Sites B1-4 provided bare control sites (background photo printed under a CC BY license, with permission from R. Orth [51]).

The restored South Bay eelgrass meadow and surrounding area is part of the Virginia Coast Reserve, a Long-Term Ecological Research site (VCR-LTER). South Bay is separated from the Atlantic Ocean by Wreck Island to the east and bordered to the west by Man and Boy Channel. The meadow bathymetry is shallow, with an average depth at MSL of 0.76 m and a standard deviation of 0.28 m [48]. Tides enter and exit the area via inlets to the north and south of the meadow. The mean tidal range is 1.2 m (Fig 2) [46]. The Virginia coastal bays are oligotrophic [49], resulting in a light environment conducive for plant growth [42,50]. Shoot densities exceed 600 shoots m^-2^ at maximum biomass in the summer, and the canopy height ranges from 32–45 cm [42]. The east half of the meadow adjoins Wreck Island; the west half of the meadow has a well-defined edge that separates the meadow from bare, subtidal areas (Fig 2). South Bay’s large surface area, relatively constant bathymetry, and a water depth to canopy height ratio of approximately 2:1 at MSL make it an ideal location for assessing canopy-hydrodynamic effects. Restoration has resulted in increased sediment carbon concentrations in the meadow [42], such that a mid-meadow site exhibited significantly higher carbon content than an adjacent bare site in 2012, 0.52±0.010% versus 0.36±0.012% [3]. Hansen and Reidenbach [30,47] measured bed shear stress and suspended sediment concentrations at locations both inside and outside of this meadow from 2010 to 2011. Average Reynolds stresses were lowest in the southwest part of the meadow, approximately 0.5 cm^2^ s^-2^ (z = 0.5 m), compared with ~1 cm^2^ s^-2^ at a mid-meadow site and 1.5 cm^2^ s^-2^ outside of the meadow [30]. Suspended sediment concentrations averaged ~20 mg l^-1^ at a mid-meadow site and ~30 mg l^-1^ outside of the meadow [47].

### Data collection and sample preparation

Sediment samples were collected along eight subtidal transects in July 2013 to quantify C_org_ distribution patterns in the meadow (Fig 2). Parallel transects were laid out from meadow edge to interior in each cardinal direction to provide broad meadow coverage. The transect sites were arrayed systematically (note that permits were not required for sediment collection from public bottomlands). Each transect contained eight sites spaced 150 m apart with the exception of transect 1, which had ten sites. Eight sites fell within original restoration seed plots [40] and the others represented meadow ages ranging from <1 to 12 years due to natural meadow expansion. Four sites near the meadow provided a bare control group (Fig 2). Four replicate 60 cc hand cores were collected at each site to a depth of 12 cm and divided into four 3-cm intervals to generate a sediment C_org_ profile for each site. The bed has aggraded ~3–4 cm due to restoration [3]. Each of the 264 cores collected during this study were visually inspected for compaction when collected, which was approximately 7%, given the predominantly sandy sediment in this study area (mean grain size = 71 μm [45]).

Macroscopic roots, rhizomes (i.e. belowground biomass), and shell fragments were removed from samples to isolate the sediment organic matter (OM) component from belowground biomass. Sediment bulk density, %OM, and percent carbon and nitrogen (%C and %N) were determined following standard methods used previously at this site [3,42]. Loss on ignition (LOI) in a muffle furnace at 500°C for six hours was used to determine %OM. A Carlo Erba NA 2500 Elemental Analyzer was used to determine %C and %N. Bulk C_org_ was determined using the element analyzer method described in [20].

Meadow age at each site was established using aerial photographs taken annually beginning in 2001 (high resolution images provided by R. Orth, VIMS [51]). Sample sites were georeferenced relative to each aerial photo in ArcGIS 10.2. By observing meadow presence/absence in each photo, we determined the length of time that seagrass has been present with one-year precision. The original seed plots coalesced over time into a single meadow, which continued to expand naturally, such that seagrass remained present at every meadow site after its first appearance (Fig 2).

The georeferenced aerial photographs also allowed us to determine site distance from the meadow perimeter. Site edge proximity was measured two different ways: linear distance along transects to the 2013 meadow perimeter and Euclidean distance to the 2013 edge (Near analysis in ArcGIS 10.2). The first measure allowed us to compare C_org_ concentration changes with distance along a given transect. The second measure established site location relative to the meadow boundary that intercepts incoming current and wave energy.

Several additional variables were also measured that could influence the C_org_ spatial distribution pattern, including site relative exposure, peak-summer (July) shoot density (shoots m^-2^), sediment grain size distribution (top 3 cm of the bed), mean water depth (bathymetry), and sediment C:N ratio. A relative exposure index (REI) was calculated for every site according to methods in [21]. Effective fetch was found by intersecting radiating lines at each site with surrounding land surfaces delineated using aerial imagery in ArcGIS 10.2. Wind vector and frequency data necessary for REI was obtained from a LTER monitoring station immediately south of the meadow on Godwin Island [52]. Replicate shoot density counts and grain size samples were collected at a subset of sites that were randomly selected to provide broad spatial coverage (n = 16). Average density counts were also taken at six additional, mid-meadow sites during a VCR-LTER annual survey. Shell fragments were removed from grain size samples, which were oxidized using 30% H_2_0_2_ and then acidified using a 5.0 pH acetic acid and NaOAc solution to remove OM and carbonates. Grain size distributions were determined using a Beckman-Coulter BLS 13 320 Laser Diffraction Particle Size Analyzer. Water depth at each site was determined by extracting bathymetric data [48] by site in ArcGIS 10.2. REI and bathymetry provided measures of relative wave energy and tidal current strength, respectively [53]. Mean grain size provided a measure of time-integrated shear stress and water residence time [54,55]. The C:N ratio provided an indication of OM source.

### Analyses

We identified meadow-scale spatial patterns by kriging sediment %OM, bulk C_org_, bulk C:N, shoot density, and grain size (mean grain size and % sand fraction) in ArcGIS 10.2 Geostatistical Analyst. Kriging accounted for spatial autocorrelation and provided error estimates for interpolated values (cf. [56] and references therein), which allowed us to generate robust distribution maps. We fit multiple variogram models to each dataset (stable, circular, spherical, exponential, and Gaussian) and cross-validated the models using root mean square errors. Summing the kriged C_org_ distributions allowed us to quantify the gross C_org_ stock to a depth of 12 cm. We subtracted the average background concentration from each map cell by bed interval to assess the enhanced (i.e. net) stock attributable to the meadow.

We evaluated observed gradient relationships between C_org_, meadow age, and edge proximity using regression analysis (lm, stats package) and Kendall correlation (rcor.test, ltm package) in R [57] to determine whether the variables met multiple regression assumptions. Correlation analysis allowed us to check for possible multi-collinearity between age and edge proximity (Euclidean distance). Linear regression analyses allowed us to determine whether variable relationships exhibited linearity—a multiple regression assumption. Shapiro-Wilks tests were run on C_org_ concentrations by depth interval (shapiro.test, stats package) to verify that the response variables were normally distributed. In addition to age and edge proximity, our primary variables of interest, we also considered gradient relationships between C_org_ and several potential explanatory variables: REI, density, mean grain size, and bathymetry. The Kendall correlation analysis included kriged values for density and grain size, in addition to measured values, to obtain equal sample sizes for all six independent variables.

Spatial autocorrelation potentially confounds attempts to determine the relative importance of age and edge proximity in predicting sediment C_org_ concentrations at the meadow-scale. To address this, we compared meadow age and edge proximity (Euclidean distance) effects on sediment C_org_ using spatial autoregressive models (spdep package version 0.6–4 for [57]), which utilized a neighborhood weights matrix and Moran’s *I* to account for autocorrelation (for example, [58]). We evaluated the C_org_ data for both the 0–3 cm and 3–6 cm depth intervals. The top ~3 cm of the bed accumulated after the start of the restoration [3]. The 3–6 cm interval approximately corresponded with the rhizosphere in this system. Spatial lag and error dependencies were evaluated using Lagrange Multiplier tests (lm.LMtests, spdep package, see [59]). The best models were determined by maximum likelihood estimation using Akaike’s Information Criterion (AIC).

## Results

### Spatial distribution of sediment C_org_

**Fig 3:**
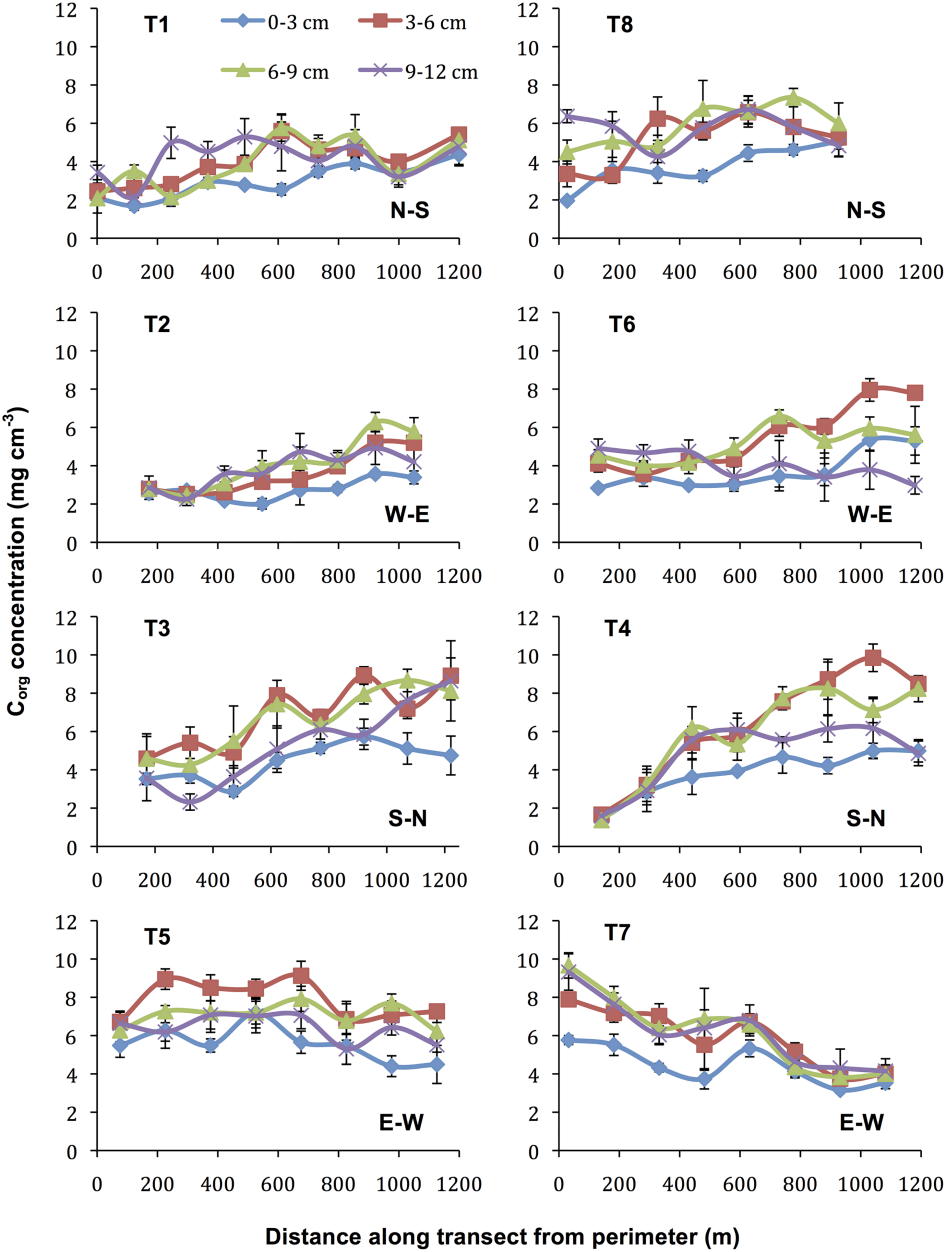
Sediment C_org_ concentrations along transects by bed depth interval. Error bars = SE.

Sediment C_org_ concentrations varied across the meadow and with sediment depth. The meadow-wide gross concentration for the top three cm of the bed averaged 3.92±0.15 (SE) mg C_org_ cm^-3^; the 3–6 cm bed depth interval had the highest average concentration, 5.66±0.25 (SE) mg C_org_ cm^-3^ (Table 1). The gross concentration ranged from 1.42 to 7.19 mg C_org_ cm^-3^ in the top three cm and from 1.66 to 9.84 mg C_org_ cm^-3^ in the 3–6 cm interval. However, this variability was non-randomly distributed across the meadow. C_org_ concentrations showed significant spatial autocorrelation at all distances ≤ 1160 m for all bed depth intervals (Moran’s *I* > 0.04, *p* < 0.05), resulting in strong spatial gradients. Sediment C_org_ decreased along each of the six transects extending from the meadow interior to the edge (Fig 3 and Table 2). In contrast, C_org_ concentrations increased with distance from the meadow interior to the perimeter on the two transects adjacent to the barrier island (T5 and T7 in Fig 3).

**Table 1:** Sediment C_org_ concentrations and blue carbon stocks within the seagrass meadow by bed depth interval (CO_2_ estimated using molecular weight ratio).

		0–3 cm	3–6 cm	6–9 cm	9–12 cm
**Bare concentration (mg cm**^**-3**^**)**	Mean	2.53	3.53	4.09	4.87
	*S*	0.38	0.94	1.81	1.28
	SE	0.22	0.54	1.04	0.74
**Meadow concentration (mg cm**^**-3**^**)**	Mean	3.92	5.66	5.60	5.04
	*S*	1.23	2.03	1.80	1.57
	SE	0.15	0.25	0.22	0.20
**Meadow stocks (t)**	Gross C_org_	706.50	1051.84	1007.63	896.01
	Net C_org_	320.14	447.56	304.85	100.61
	Net CO_2_	1173.85	1641.06	1117.79	368.92

**Table 2:** Linear relationships between sediment C_org_ and independent variables measured at sites by bed depth interval.

	n	Intercept±SE	M±SE	F (df)	*p*	adj-R^2^
Transect Dist. (0–3 cm)	48	2.53±0.329	0.ȗ±4.64E-4	21.89 (1,46)	2.56E-05	0.308
Transect Dist. (3–6 cm)	48	3.30±0.548	0.00387±7.74E-4	25.03 (1,46)	8.72E-06	0.338
Transect Dist. (6–9 cm)	48	3.47±0.463	3.22E-3±6.54E-4	24.29 (1,46)	1.12E-05	0.331
Transect Dist. (9–12 cm)	48	3.36±0.415	2.26E-3±5.86E-4	14.86 (1,46)	3.57E-04	0.228
Euclid. Dist. (0–3 cm)	64	2.20±0.243	3.04E-3±3.84E-4	62.78 (1,62)	5.38E-11	0.495
Euclid. Dist. (3–6 cm)	64	2.81±0.401	5.02E-3±6.34E-4	62.88 (1,62)	5.24E-11	0.496
Euclid. Dist. (6–9 cm)	64	3.45±0.400	3.81E-3±6.32E-4	36.29 (1,62)	1.01E-07	0.359
Euclid. Dist. (9–12 cm)	64	3.74±0.398	2.31E-3±6.29E-4	13.42 (1,62)	5.18E-04	0.165
Age (0–3 cm)	66	2.84±0.192	0.192±4.68E-2	16.83 (1,64)	1.12E-04	0.196
Age (3–6 cm)	66	3.96±0.480	0.301±7.83E-2	14.81 (1,64)	2.77E-04	0.175
LogAge (0–3 cm)*	66	0.29±0.050	0.366±6.39E-2	32.91 (1,64)	2.84E-07	0.340
LogAge (3–6 cm)*	66	0.40±0.060	0.412±7.66E-2	28.95 (1,64)	1.13E-06	0.311
REI (0–3 cm)	66	8.68±1.30	-1.56E-6±4.19E-7	13.75 (1,64)	4.39E-04	0.164
REI (3–6 cm)	66	13.5±2.15	-2.58E-6±6.91E-7	13.92 (1,64)	4.08E-04	0.166
Density (0–3 cm)	16	2.37±0.835	3.64E-3±1.68E-3	4.687 (1,14)	4.82E-02	0.197
Density (3–6 cm)	16	4.43±1.29	3.60E-3±2.60E-3	1.919 (1,14)	1.88E-01	0.058
Grain size (0–3 cm)	16	9.72±0.939	-6.08E-2±9.93E-3	37.51 (1,14)	2.63E-05	0.709
Grain size (3–6 cm)	16	14.6±1.11	-9.17E-2±1.17E-2	61.1 (1,14)	1.79E-06	0.800

*Regressions were run on log-transformed C_org_ data

**Fig 4:**
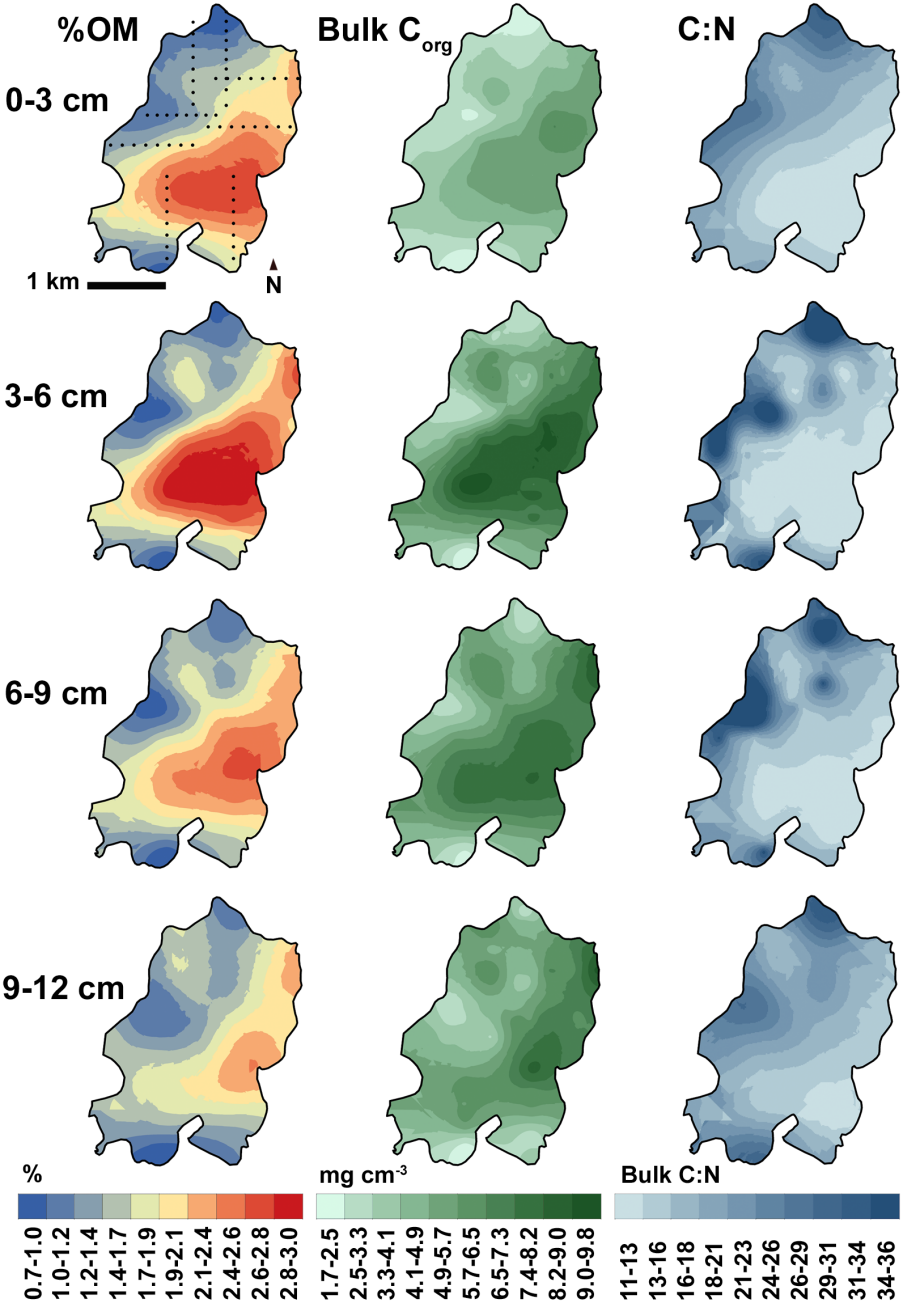
Organic matter, C_org_, and bulk C:N distributions by bed depth interval within the meadow. Transect sites are shown in the first Fig; the maps were generated by kriging.

**Fig 5:**
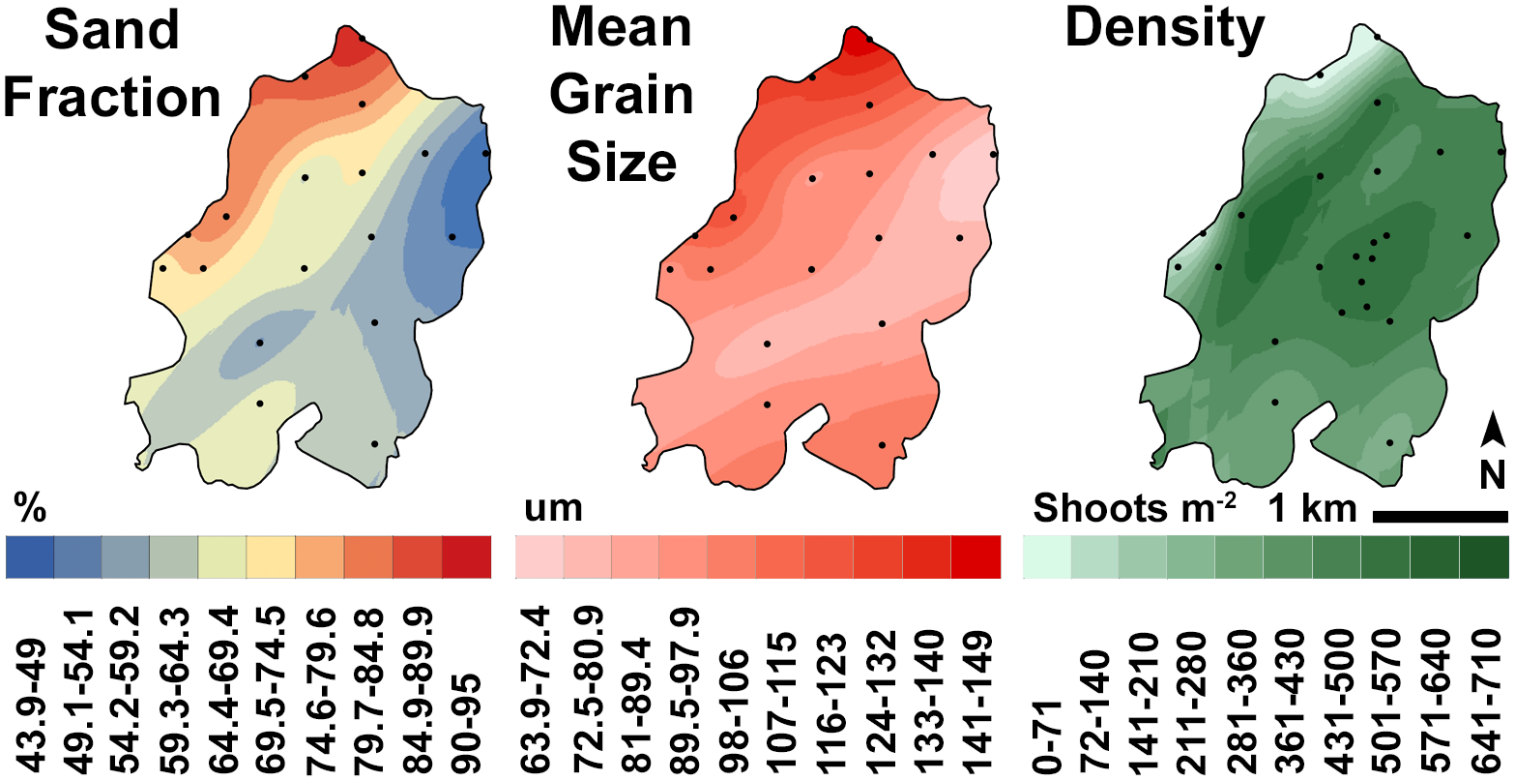
Meadow grain size (mean and sand fraction) and peak seagrass shoot density distributions. Sample sites are shown in each Fig; the maps were generated by kriging. Note that the inverse of sand fraction represents < sand-size particles.

Interpolating the %OM, bulk C_org_, and C:N data consistently yielded two discrete spatial regimes: a kriged zone of higher %OM and C_org_ encompassing most of the southeastern half of the meadow and another zone of decreasing %OM and C_org_ to the northwest (Fig 4). C:N ratios and grain size data yielded a similar kriged pattern, with higher C:N ratios and a higher percentage of larger grains to the northwest and lower values to the southeast, near Wreck Island (Figs 4 and 5). Summing the interpolated sediment C_org_ within the meadow area to a bed depth of 12 cm gave a total meadow (gross) stock of 3662 t C_org_. Subtracting average background concentrations measured at the bare sites from each meadow site by depth interval and interpolating the net increase gave a net stock of 1173 t C_org_.

### Spatial variables

Several factors possibly account for the spatial distribution of sediment C_org_. Edge proximity, meadow age, grain size and bathymetry were all significantly correlated with C_org_ at the meadow-scale. Edge proximity regression relationships (Euclidean distance) were highly significant at all four depth intervals. The highest adjusted-r^2^ values were for the 0–3 and 3–6 cm intervals (Table 2). C_org_ and meadow age were highly correlated, but exhibited a relatively weak, positive linear regression relationship (Tables 2 and 3). The strongest regression and correlation relationships were between the 0–3 cm C_org_ and sediment grain size (Tables 2 and 3). C_org_ concentrations were not significantly correlated with shoot density or REI (Table 3). Unlike the kriged grain size distributions, the kriged density distribution did not match the C_org_ distribution (Fig 5).

**Table 3:** Kendall correlation (Tau B) for sediment bulk C_org_ and possible explanatory variables (n = 66 sites; top number = τ, bottom number = *p*-value; significant correlations at Bonferroni adjusted α’<0.0018 highlighted in bold).

	**0–3 C**_**org**_	**3–6 C**_**org**_	**Euclid. Dist.**	**Age**	**REI**	**Density**	**Grain size**	**Bathymetry**
**0–3 C**_**org**_	*****	0.692	0.534	0.396	-0.249	0.117	-0.605	0.322
**3–6 C**_**org**_	**<0.001**	*****	0.517	0.376	-0.264	0.113	-0.56	0.294
**Euclid. Dist.**	**<0.001**	**<0.001**	*****	0.574	-0.186	0.289	-0.575	0.277
**Age**	**<0.001**	**<0.001**	**<0.001**	*****	-0.057	0.382	-0.45	0.121
**REI**	0.003	0.002	0.027	0.524	*****	0.067	0.29	-0.379
**Density**	0.165	0.179	**0.001**	**<0.001**	0.429	*****	-0.192	-0.037
**Grain Size**	**<0.001**	**<0.001**	**<0.001**	**<0.001**	**0.001**	0.023	*****	-0.339
**Bathymetry**	**<0.001**	**<0.001**	**0.001**	0.174	**<0.001**	0.662	**<0.001**	*****

Edge proximity, age, density, grain size, and bathymetry were also significantly correlated with one another, potentially indicative of landscape-scale interactions among the variables. Grain size was negatively correlated with edge proximity, age, and bathymetry, and positively correlated with REI (Table 3). The strongest correlation coefficient was between grain size and site distance from the open perimeter (τ = -0.575). Site age and edge proximity (Euclidean distance) were also moderately correlated but not co-variates (τ = 0.574), allowing us to compare their ability to predict C_org_ concentrations using multiple regression. The meadow has expanded outward over time, but it also coalesced in places and filled in behind Wreck Island relatively recently (Fig 2).

**Fig 6:**
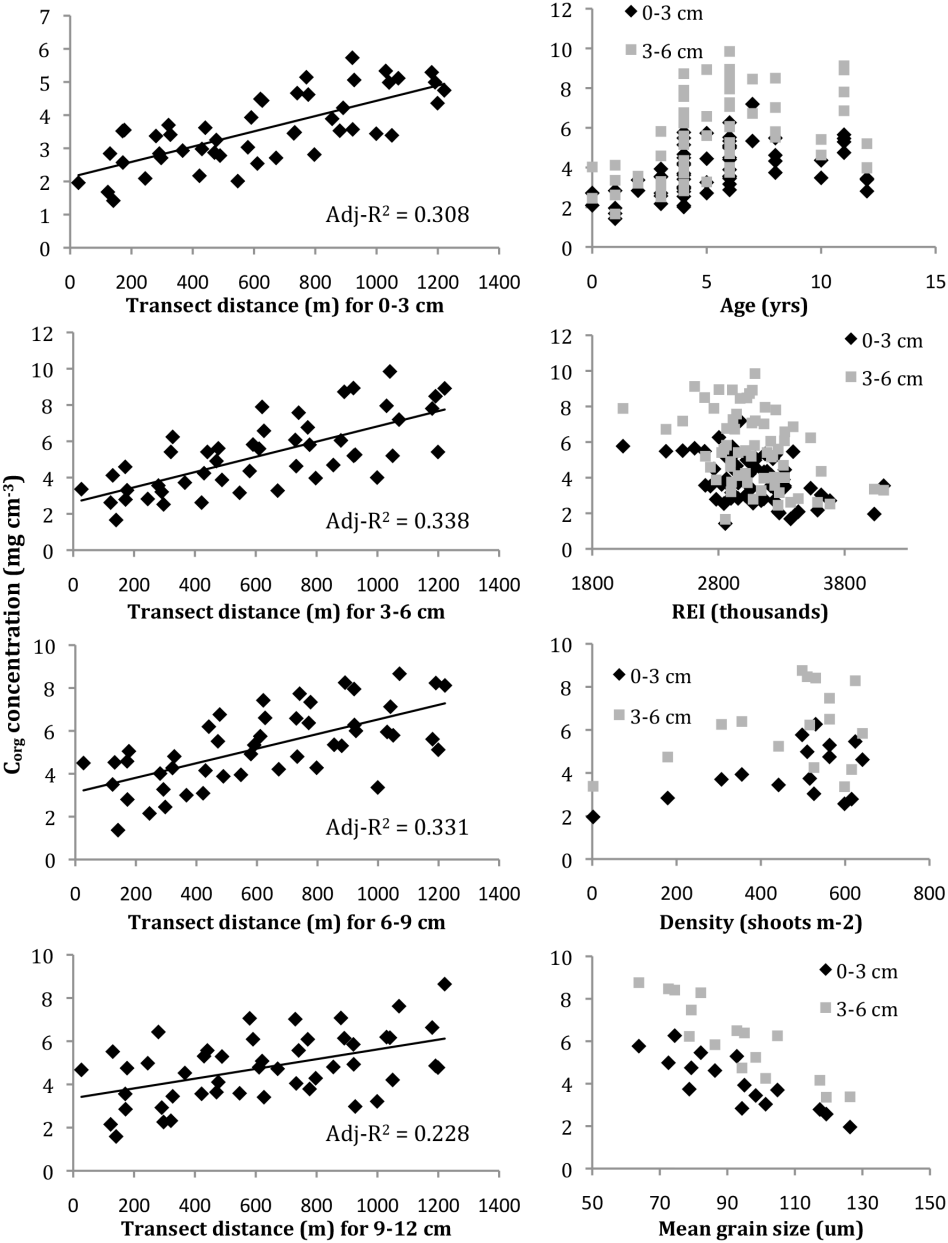
Sediment C_org_ concentration relationships with measured independent variables. Comparisons are by bed depth interval as noted; see Table 2 for individual regression statistics.

Several factors complicated our ability to compare the effects of edge proximity and age on C_org_ concentrations directly through multiple regression. C_org_ varied non-linearly with age (Fig 6), so we applied a log-log transformation, which yielded linear relationships with adj-r^2^ values >0.3 for both the 0–3 and 3–6 cm data (Table 2). The log-transformed C_org_ data for the 0–3 cm interval met the dependent variable normality assumption (W = 0.972, *p* = 0.143) and also varied linearly with the edge data. The log-transformed C_org_ data for the 3–6 cm interval was not normally distributed (W = 0.963, *p* = 0.044). Running a multiple regression analysis comparing edge and log-transformed age on C_org_ concentrations within the top three cm of the bed yielded spatially autocorrelated regression residuals (Moran’s *I* = 0.535, *p*<1.10E-4). Lagrange Multiplier tests found strong spatial lag dependence (RLMlag = 6.1796, *p*<0.013). We accounted for this spatial autocorrelation using spatial lag and error analyses, both of which identified edge proximity—not age—as a significant predictor of C_org_ concentrations at the meadow-scale (Table 4).

**Table 4:** Spatial autoregressive model results for 0–3 cm data.

	LogC_org_ 0–3 cm Spatial Autoregressive Lag Model			
	Coefficient	SE	Z	Probability
(Intercept)	2.26E-01	5.01E-02	4.52	6.27E-06
LogAge	4.76E-02	6.86E-02	0.693	0.488
Edge	1.91E-04	5.90E-05	3.24	1.21E-03
Z	4.40	0.081*		1.10E-05
rho	0.355			1.64E-04
AIC	-118			
	LogC_org_ 0–3 cm Spatial Autoregressive Error Model			
	Coefficient	SE	Z	Probability
(Intercept)	4.03E-01	4.75E-02	8.48	< 2.2e-16
LogAge	-5.95E-03	8.18E-02	-0.073	0.942
Edge	3.09E-04	7.42E-05	4.17	3.10E-05
Z	4.13	0.083*		3.57E-05
λ	0.345			9.53E-04
AIC	-114			

*Asymptotic standard error

## Discussion

### Meadow-scale controls on sediment C_org_ accumulation

Our results indicate that differences in relative location within the meadow affect the C_org_ stock distribution and overshadow other factors, including seagrass age and shoot density, that are known to affect C_org_ concentrations at the plot-scale. We show that edge proximity affects C_org_ concentrations over much larger spatial scales than previous studies have recognized, potentially resulting in seagrass meadow spatial gradients >1 km in length. Carbon stock estimates should take these potential meadow-scale spatial patterns into account.

Rather than reflecting the meadow’s expansion history over the preceding 12-year period, the meadow-wide C_org_ distribution appears broadly consistent with the hypothesis that current attenuation promotes higher C_org_ concentrations with distance from the edge (Fig 1A). The meadow-wide grain size distribution (Fig 4) also supports this hypothesis. Previous studies have noted that suspended sediment is advected into this meadow [30], increasing the percentage of silts and fine sands at meadow sites over time [42]. This suspended sediment fractionates according to particle size as it is deposited across the meadow, with finer particles settling out in the southeastern meadow, where Hansen and Reidenbach [30] documented the lowest average Reynolds stress. This, in turn, facilitates more C_org_ storage, because smaller grains have more surface area for C_org_ adsorption [55]. This process appears to be driven largely by tidal currents. Hansen and Reidenbach [30] observed similar Reynolds stresses attributable to wave-dominated flows in different meadow locations, which may explain why we did not observe a significant correlation between C_org_ and REI. In addition to canopy current attenuation, basin geomorphology possibly accounts for some of the reduction in current velocity in the area of the meadow adjacent to Wreck Island and furthest from the two inlets. We note that a C_org_ spatial pattern is weakly present within the underlying 9–12 cm depth interval (Fig 4), which was deposited prior to the meadow restoration [3]. However, the meadow has clearly accentuated the observed pattern, as evidenced by the magnitude of the discrepancy in C_org_ concentrations between the two spatial regimes within the top 6 cm of the bed (Fig 4). Root and rhizome-derived carbon compounds may also contribute to sediment C_org_ accumulation below 6 cm.

These results confirm that large, sediment C_org_ spatial gradients are possible and should be considered when estimating blue carbon stocks. Similar studies are now needed to determine how varying current velocity, meadow configuration, and the water depth to canopy height ratio might give rise to particular sediment C_org_ gradients at this spatial scale. Other C_org_ distributions may be possible in other meadows. For example, an even larger meadow with a lower water depth to canopy height ratio might experience lower C_org_ concentrations further from the edge, because of reduced sediment delivery (Fig 1C). However, given that we observed increasing sediment C_org_ concentration gradients (Fig 1A) >1 km in length, and given that smaller, patchy meadows are relatively common, edge proximity possibly limits C_org_ accumulation in many systems. Other studies have speculated along these lines but lacked the ability to control for meadow expansion as a possible confounding variable (for example, [27]). Our results also highlight the importance of considering spatial autocorrelation and its potential effect on measured quantities at individual sites within a given seagrass meadow.

The potential importance of external sediment raises the possibility that much of the sediment C_org_ stored in this meadow is, in fact, allochthonous in origin. Greiner et al. [44] found that only half of the sediment C_org_ at an interior site in the South Bay meadow derived from vascular plants. The C:N ratio in the C_org_ hotspot in the southeast part of the meadow (Fig 4) conforms more closely to the range observed for seston and macroalgae than for *Z*. *marina* in this system [60]. The high C:N values in the northwest half of the meadow more closely resemble *Z*. *marina*. The grain size spatial distribution suggests that the southeastern meadow experiences lower current velocities and longer residence times, both of which possibly increase seston accumulation, which would increase the magnitude of observed C_org_ spatial gradients across the meadow. However, additional isotopic work is needed to conclusively identify C_org_ sources at this spatial scale.

The fact that edge proximity, not age, significantly predicts meadow-wide sediment C_org_ concentrations indicates that meadow- and regional-scale factors should be considered when estimating whole-meadow carbon stocks. Recent studies consider blue carbon accumulation as a function of plot-scale factors, including meadow age and plant density (for example, [61]), without considering possible spatial scale effects. Age and C_org_ concentrations are positively correlated at individual sites in this study, but differences attributable to relative location overshadow differences due to age at this spatial scale. Shoot density also affects sediment C_org_ accumulation at the plot-scale [42,62], but density alone is not a good proxy for site C_org_ concentrations at the meadow-scale. This is likely due to the fact that meadow canopy structure (i.e. shoot density and biomass) varies considerably over small spatial and short temporal scales [42,63] and because density effects on sediment resuspension appear to be non-linear [45]. Consequently, a snapshot assessment of canopy structure would not necessarily correspond with the sediment C_org_ distribution, which reflects the balance of accumulation and resuspension over interannual timescales. Likewise, REI might correlate with C_org_ in isolated seagrass patches but does not account for current attenuation by the canopy.

### Implications for financing seagrass restoration using blue carbon offset-credits

The distribution of sediment C_org_ in this meadow follows approximately linear C_org_ concentration gradients—not irregular zones of higher and lower C_org_ concentrations controlled by wind fetch, canopy complexity, age, or other factors. A representative C_org_ concentration for stock estimation might, therefore, be obtained by averaging samples collected from a relatively small number of sites distributed along the gradient. However, managers should avoid overestimating C_org_ stocks by relying on point-based literature values, models, or default values—all permissible approaches under VM0033 [38]. Scaling the C_org_ measurement reported for this meadow by Greiner et al. [3] by the total meadow area would overestimate the gross sediment C_org_ stock by almost 20%, because of the spatial gradients. Likewise, managers should not simply scale C_org_ accumulation model results calculated for small unit areas (for example, [61]) or rely exclusively on near-surface ^210^Pb accumulation rates that do not account for remineralization [64], without understanding possible meadow-scale effects.

Regarding carbon offset-credit finance for seagrass restoration, the enhanced sediment C_org_ stock attributable to the meadow after more than a decade translates to approximately 4,300 t CO_2_. Incorporating sequestered *Z*. *marina* biomass C_org_ would increase this total [12]. However, the aboveground biomass is sloughed off, and the fate of this exported C_org_ is uncertain, so the sequestered stock would correspond to the annual cycle average, not peak standing biomass.

Managers might be able to increase blue carbon storage by considering meadow configuration, basin geomorphology, and regional hydrodynamics when locating seagrass restoration sites. A large, contiguous meadow should store more blue carbon than an equal area of small meadow patches. If blue carbon storage is a management goal, restoration should be initiated at sites that are suitable for the accumulation of fine sediment. As the meadow expands, these locations should accumulate more blue carbon, due to scale-dependencies observed in this study, and adjacent areas should begin to bury blue carbon, thanks to the canopy particle-trapping feedback [29,37]. Additional studies that determine how current velocity, meadow configuration, and water depth interact to influence meadow-scale C_org_ gradients can aid blue carbon accumulation modeling efforts at spatial scales relevant to restoration managers.

## Conclusions

This study indicates that edge proximity can better explain a seagrass meadow’s sediment C_org_ distribution than spatial differences in accumulation time. Although meadow age and seagrass shoot density affect C_org_ accumulation at the plot-scale, these drivers can be overshadowed by differences in relative location at the meadow-scale. Progressive canopy attenuation of currents may explain the C_org_ distribution observed in this study. As currents move through the canopy, suspended sediment becomes stratified and is deposited according to particle size, which likely facilitates more C_org_ burial at more interior sites, irrespective of site meadow age. These findings highlight the potential importance of external sediment for seagrass blue carbon accumulation and the need to consider meadow-scale spatial gradients when quantifying whole-meadow carbon stocks.

## References

[1] Duarte CM, Chiscano CL. Seagrass biomass and production: a reassessment. *Aquatic Botany*. 1999;65: 159–174.

[2] Duarte CM, Marbà N, Gacia E, Fourqurean JW, Beggins J, Barron C, et al Seagrass community metabolism: assessing the carbon sink capacity of seagrass meadows. *Global Biogeochemical Cycles*. 2010;24: GB4032.

[3] Greiner JT, McGlathery KJ, Gunnell J, McKee BA. Seagrass restoration enhances “blue carbon” sequestration in coastal waters. *PLoS ONE*. 2013;8(8): e72469 10.1371/journal.pone.0072469 23967303 PMC3743776

[4] Smith SV. Marine macrophytes as a global carbon sink. *Science*. 1981;211(4484): 838–840. 10.1126/science.211.4484.838 17740399

[5] de Boer WF. Seagrass—sediment interactions, positive feedbacks and critical thresholds for occurrence: a review. *Hydrobiologia*. 2007;591: 5–24.

[6] Carr JA, D’Odorico P, McGlathery K, Wiberg P. Stability and bistability of seagrass ecosystems in shallow coastal lagoons: Role of feedbacks with sediment resuspension and light attenuation. *Journal of Geophysical Research*. 2010;115: G03011.

[7] Duarte CM, Kennedy H, Marbà N, Hendricks I. Assessing the capacity of seagrass meadows for carbon burial: Current limitations and future strategies. *Ocean & Coastal Management*. 2013;83: 32–38.

[8] Kennedy H, Beggins J, Duarte CM, Fourqurean JW, Holmer M, Marbà N, et al Seagrass sediments as a global carbon sink: Isotopic constraints. *Global Biogeochemical Cycles*. 2010;24: GB4026.

[9] Waycott M, Duarte CM, Carruthers TJB, Orth RJ, Dennison WC, Olyarnik S, et al Accelerating loss of seagrass across the globe threatens coastal ecosystems. *Proceedings of the National Academy of Sciences*. 2009;106(30): 12377–12381. 10.1073/pnas.0905620106 PMC2707273 19587236

[10] Marbà N, Arias-Ortiz A, Masqué P, Kendrick GA, Mazarrasa I, Bastyan GR, et al Impact of seagrass loss and subsequent revegetation on carbon sequestration and stocks. *Journal of Ecology*. 2015;103: 296–302.

[11] Macreadie PI, Trevathan-Tackett SM, Skilbeck CG, Sanderman J, Curlevski N, Jacobsen G, et al Losses and recovery of organic carbon from a seagrass ecosystem following disturbance. *Proc B*. 2015;282: 20151537. 10.1098/rspb.2015.1537 PMC4633871 26490788

[12] Fourqurean JW, Duarte CM, Kennedy H, Marbà N, Holmer M, Mateo MA, et al Seagrass ecosystems as a globally significant carbon stock. *Nature Geoscience*. 2012;5: 505–509.

[13] Pendleton L, Donato DC, Murray BC, Crooks S, Jenkins WA, Sifleet S, et al Estimating global “blue carbon” emissions from conversion and degradation of vegetated coastal ecosystems. *PLoS ONE*. 2012;7(9): e43542 10.1371/journal.pone.0043542 22962585 PMC3433453

[14] Nellemann C, Corcoran E, Duarte CM, Valdés L, De Young C, Fonseca L, et al., editors. Blue Carbon: A Rapid Response Assessment. United Nations Environment Programme, GRID-Arendal; 2009.

[15] Murray BC, Pendleton L, Jenkins WA, Sifleet S. Green Payments for Blue Carbon: Economic Incentives for Protecting Threatened Coastal Habitats. Nicholas Institute for Environmental Policy Solutions Report NI R 11–04. Durham, North Carolina: Duke University; 2011.

[16] Moriarty DJW, Iverson RL, Pollard PC. Exudation of organic carbon by the seagrass *Halodule wrightii Aschers* and its effect on bacterial growth in the sediment. *Journal of Experimental Marine Biology and Ecology*. 1986;96(2): 115–126.

[17] Duarte CM, Middelburg JJ, Caraco N. Major role of marine vegetation on the oceanic carbon cycle. *Biogeosciences*. 2005;2: 1–8.

[18] Mateo MA, Cebrián J, Dunton K, Mutchler T. Carbon flux in seagrass ecosystems In: Larkum AWD, Orth RJ, Duarte C, editors. *Seagrasses*: *Biology*, *Ecology and Conservation*. Netherlands: Springer; 2006 pp. 159–192.

[19] Mcleod E, Chmura GL, Bouillon S, Salm R, Björk M, Duarte CM, et al A blueprint for blue carbon: toward an improved understanding of the role of vegetated coastal habitats in sequestering CO_2_. *Frontiers in Ecology and the Environment*. 2011;9(10): 552–560

[20] Howard J, Hoyt S, Isensee K, Pidgeon E, Telszewski M, editors. Coastal blue carbon: methods for assessing carbon stocks and emissions factors in mangroves, tidal salt marshes, and seagrass meadows. Conservation International, Intergovernmental Oceanographic Commission of UNESCO. Arlington, Virginia: IUCN; 2014.

[21] Fonseca MS, Bell SS. Influence of physical setting on seagrass landscapes near Beaufort, North Carolina, USA. *Marine Ecology Progress Series*. 1998;171: 109–121.

[22] Bell SS, Fonseca MS, Stafford NB. Seagrass ecology: New contributions from a landscape perspective In: Larkum AWD, Orth RJ, Duarte C, editors. *Seagrasses*: *Biology*, *Ecology and Conservation*. Netherlands: Springer; 2006 pp. 625–645.

[23] Granata TC, Serra T, Colomer J, Casamitjana X, Duarte CM, Gacia E. Flow and particle distributions in a nearshore seagrass meadow before and after a storm. *Marine Ecology Progress Series*. 2001;218: 95–106.

[24] Samper-Villarreal J, Lovelock CE, Saunders MI, Roelfsema C, Mumby PJ. Organic carbon in seagrass sediments is influenced by seagrass canopy complexity, turbidity, wave height, and water depth. *Limnology and Oceanography*. 2016;61: 938–952.

[25] Lavery PS, Mateo M-Á, Serrano O, Rozaimi M. Variability in the carbon storage of seagrass habitats and its implications for global estimates of blue carbon ecosystem service. *PLoS ONE*. 2013;8(9): e73748 10.1371/journal.pone.0073748 24040052 PMC3764034

[26] Serrano O, Ricart AM, Lavery PS, Mateo MA, Arias-Ortiz A, Masque P, et al Key biogeochemical factors affecting soil carbon storage in *Posidonia* meadows. *Biogeosciences Discussions*. 2015;12: 18913–18944.

[27] Ricart AM, York PH, Rasheed MA, Pérez M, Romero J, Bryant CV, et al Variability of sedimentary organic carbon in patch seagrass landscapes. *Marine Pollution Bulletin*. 2015;100(1): 476–482. 10.1016/j.marpolbul.2015.09.032 26428624

[28] López F, García M. Open-channel flow through simulated vegetation: Suspended sediment transport modeling. *Water Resources Research*. 1998;34(9): 2341–2352.

[29] Koch EW, Ackerman JD, Verduin J, van Keulen M. Fluid dynamics in seagrass ecology—from molecules to ecosystems In: Larkum AWD, Orth RJ, Duarte C, editors. *Seagrasses*: *Biology*, *Ecology and Conservation*. Netherlands: Springer; 2006 pp. 193–225.

[30] Hansen JCR, Reidenbach MA. Wave and tidally driven flows in eelgrass beds and their effect on sediment suspension. *Marine Ecology Progress Series*. 2012;448: 271–287.

[31] Mendez FJ, Losada IJ. Hydrodynamics induced by wind waves in a vegetation field. *Journal of Geophysical Research*. 1999;104(C8): 18383–18396.

[32] Chen S-N, Sanfod LP, Koch EW, Shi F, North EW. A nearshore model to investigate the effects of seagrass bed geometry on wave attenuation and suspended sediment transport. *Estuaries and Coasts*. 2007;30(2): 296–310.

[33] Lefebvre A, Thompson CEL, Amos CL. Influence of *Zostera marina* canopies on unidirectional flow, hydraulic roughenss and sediment movement. *Continental and Shelf Research*. 2010;30: 1783–1794.

[34] Carr JA, D’Odorico P, McGlathery KJ, Wiberg PL. Spatially explicit feedbacks between seagrass meadow structure, sediment and light: Habitat suitability for seagrass growth. *Advances in Water Resources*. 2015;000: 1–11.

[35] Hendriks IE, Sintes T, Bouma TJ, Duarte CM. Experimental assessment and modeling evaluation of the effects of the seagrass *Posidonia oceanica* on flow and particle trapping. *Marine Ecology Progress Series*. 2008;356: 163–173.

[36] Bradley K, Houser C. Relative velocity of seagrass blades: Implications for wave attenuation in low-energy environments. *Journal of Geophysical Research*. 2009;114: F01004.

[37] Gruber RK, Kemp WM. Feedback effects in a coastal canopy-forming submersed plant bed. *Limnology and Oceanography*. 2010;55(6): 2285–2298.

[38] Emmer I, Needelman B, Emmett-Mattox S, Crooks S, Megonigal P, Myers D, et al. Methodology for Tidal Wetland and Seagrass Restoration. Verified Carbon Standard. VM0033 Version 1.0. 2015. http://database.v-c-s.org/methodologies/methodology-tidal-wetland-and-seagrass-restoration-v10.

[39] Verified Carbon Standard. Methods for stratification of the project area (X-STR). VMD0016. 2015. http://database.v-c-s.org/sites/vcs.benfredaconsulting.com/files/VMD0016%20X-STR,%20v1.0%20(valid%20until%209%20September%202015).pdf

[40] Orth RJ, Luckenbach ML, Marion SR, Moore KA, Wilcox DJ. Seagrass recovery in the Delmarva Coastal Bays, USA. *Aquatic Botany*. 2006;84: 26–36.

[41] Orth RJ, Marion SR, Moore KA, Wilcox DJ. Eelgrass (*Zostera marina* L.) in the Chesapeake Bay Region of Mid-Atlantic Coast of the USA: Challenges in Conservation and Restoration. *Estuaries and Coasts*. 2010;33: 139–150.

[42] McGlathery KJ, Reynolds LK, Cole LW, Orth RJ, Marion SR, Schwarzschild A. Recovery trajectories during state change from bare sediment to eelgrass dominance. *Marine Ecology Progress Series*. 2012;448: 209–221.

[43] Orth RJ, Moore KA, Marion SR, Wilcox DJ, Parrish DB. Seed addition facilitates eelgrass recovery in a coastal bay system. *Marine Ecology Progress Series*. 2012;448: 177–195.

[44] Greiner JT, Wilkinson GM, McGlathery KJ, Emery KA. Sources of sediment carbon sequestered in restored seagrass meadows. *Marine Ecology Progress Series*. 2016;551: 95–105.

[45] Lawson SE, McGlathery KJ, Wiberg PL. Enhancement of sediment suspension and nutrient flux by benthic macrophytes at low biomass. *Marine Ecology Progress Series*. 2012;448: 259–270.

[46] Fagherazzi S, Wiberg PL. Importance of wind conditions, fetch, and water levels on wave-generated shear stresses in shallow intertidal basins. *Journal of Geophysical Research*. 2009;114: F03022.

[47] Hansen JCR, Reidenbach MA. Seasonal growth and senescence of a *Zostera marina* seagrass meadow alters wave-dominated flow and sediment suspension within a coastal bay. *Estuaries and Coasts*. 2013;36: 1099–1114.

[48] Richardson D, Porter J, Oertel G, Zimmerman R, Carlson C, Overman K. Integrated topography and bathymetry for the Eastern Shore of Virginia; 2014. [cited 2016 January 12]. Database: LTER Network [Internet]. 10.6073/pasta/0d07604a03d09e327abbe2b81e44ac11.

[49] McGlathery KJ, Sundback K, Anderson IC. Eutrophication in the shallow coastal bays and lagoons: the role of plants in the coastal filter. *Marine Ecology Progress Series*. 2007;348: 1–18.

[50] Moore KA, Shields EC, Parrish DB, Orth RJ. Eelgrass survival in two contrasting systems: role of turbidity and summer water temperatures. *Marine Ecology Progress Series*. 2012;448: 247–258.

[51] SAV in Chesapeake Bay and Coastal Bays; 2014 [cited 2014 November 19]. Database: Maps and Data [Internet]. http://web.vims.edu/bio/sav/.

[52] Reidenbach M, Timmerman R. Wind speed and direction on Godwin Island, 2013–2014; 2014 [cited 2016 February 24]. Database: LTER Network [Internet]. 10.6073/pasta/0d07604a03d09e327abbe2b81e44ac11.

[53] Mariotti G, Fagherazzi S. Modeling the effect of tides and waves on benthic biofilms. *Journal of Geophysical Research*. 2012;117: G04010.

[54] Lawson SE, Wiberg PL, McGlathery KJ, Fugate DC. Wind-driven sediment suspension controls light availability in a shallow coastal lagoon. *Estuaries and Coasts*. 2007;30: 102–112.

[55] Wiberg PL, Carr JA, Safak I, Anutaliya A. Quantifying the distribution and influence of non-uniform bed properties in shallow coastal bays. *Limnology and Oecanography*: *Methods*. 2015;13: 746–762.

[56] Zupo V, Mazzella L, Buia MC, Gambi MC, Lorenti M, Scipione MB, et al A Small-scale analysis of the spatial structure of a Posidonia oceanic meadow off the Island of Ischia (Gulf of Naples, Italy): Relationship with the seafloor morphology. *Aquatic Botany*. 2006;84: 101–109.

[57] R Project for Statistical Computing. R 3.2.1. 2014. https://cran.r-project.org.

[58] Genovesi B, Mouillot D, Laugier T, Fiandrino A, Laabir M, Vaquer A, et al Influences of sedimentation and hydrodynamics on the spatial distribution of *Alexandrium catenella*/*tamarense* resting cysts in a shellfish farming lagoon impacted by toxic blooms. *Harmful Algae*. 2013;25: 15–25.

[59] Zhang X, Tang D, Li Z, Yan Z, Zhang F. Analysis of the spatio-temporal distribution of chlorophyll-α in the eastern Indian Ocean near the time of the 2004 South Asian tsunami. *International Journal of Remote Sensing*. 2010;31(17–18): 4579–4593.

[60] Greiner JT. Assessing the importance of seagrass habitat restoration to “blue carbon” sequestration in shallow coastal waters. Ms Thesis, University of Virginia. 2013. http://search.lib.virginia.edu/catalog/u6124019.

[61] Duarte CM, Sintes T, Marbà N. Assessing the CO_2_ capture potential of seagrass restoration projects. *Journal of Applied Ecology*. 2013;50(6): 1341–1349.

[62] Widdows J, Pope ND, Brinsley MD, Asmus H, Asmus RM. Effects of seagrass beds (*Zostera noltii and Z*. *marina*) on near-bed hydrodynamics and sediment resuspension. *Marine Ecology Progress Series*. 2008;358: 125–136.

[63] McGlathery K. Above- and below-ground biomass and canopy height of seagrass in Hog Island Bay, VA 2007–2013; 2013 [cited 2016 July 25]. Database: LTER Network [Internet]. https://portal.lternet.edu/nis/mapbrowse?packageid=knb-lter-vcr.183.13.

[64] Johannessen SC, Macdonald RW. Geoengineering with seagrasses: is credit due where credit is given? *Environmental Research Letters*. 2016;11: 113001.

